# Supplementary figures and images for: Endothelial MerTK impairment accelerates the development of atherosclerosis
Source: Redox Biol. 2025 Sep 11;87:103861. doi: 10.1016/j.redox.2025.103861 (PMC12628026; doi:10.1016/j.redox.2025.103861)

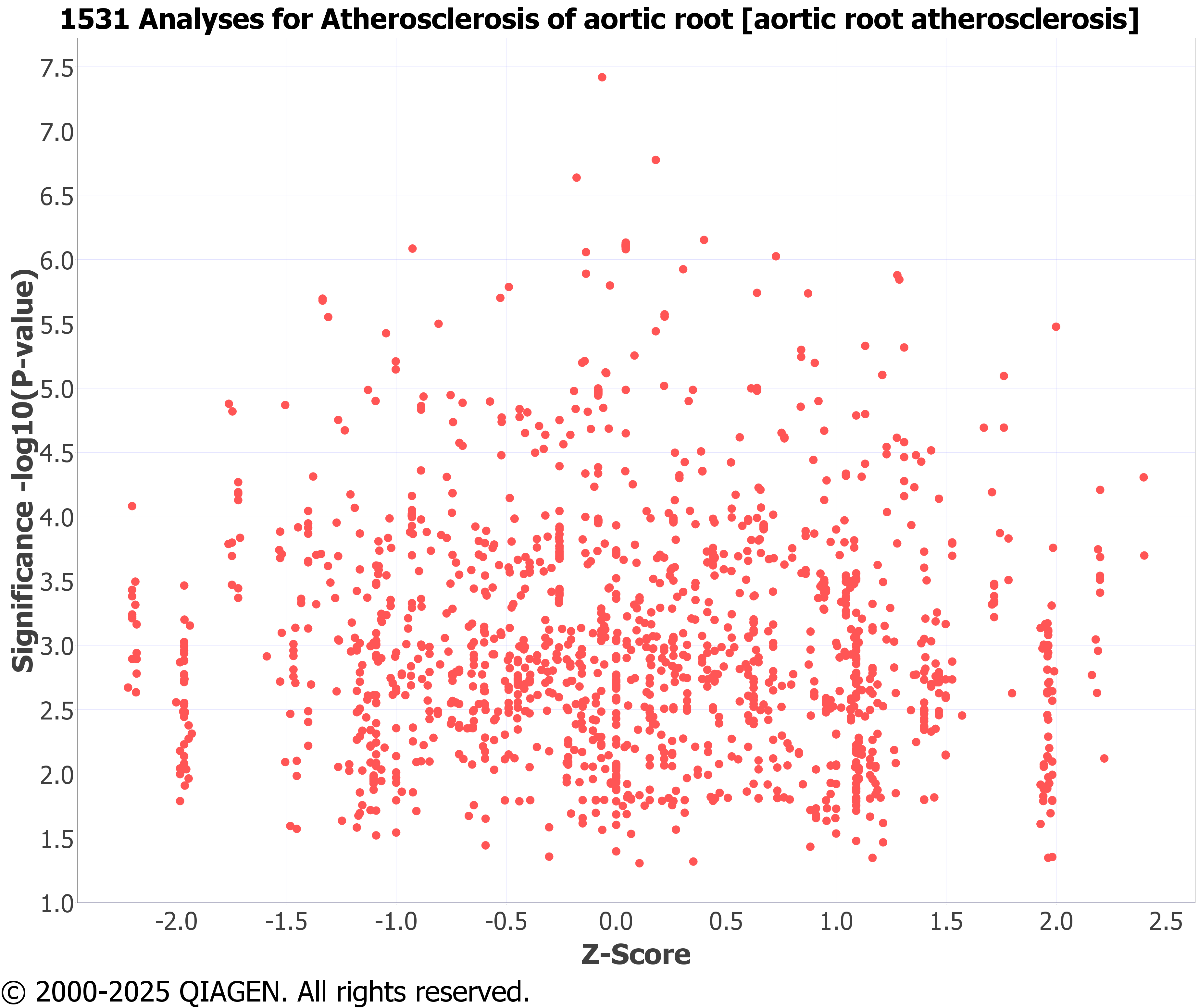

Supplement: Multimedia component 1 [file mmc1.zip › Supplement data/IPA Big data analytics-Atherosclerosis overall signaling and RNA-seq for MerTK expression/1531 analyses for AS in aortic root.tif]

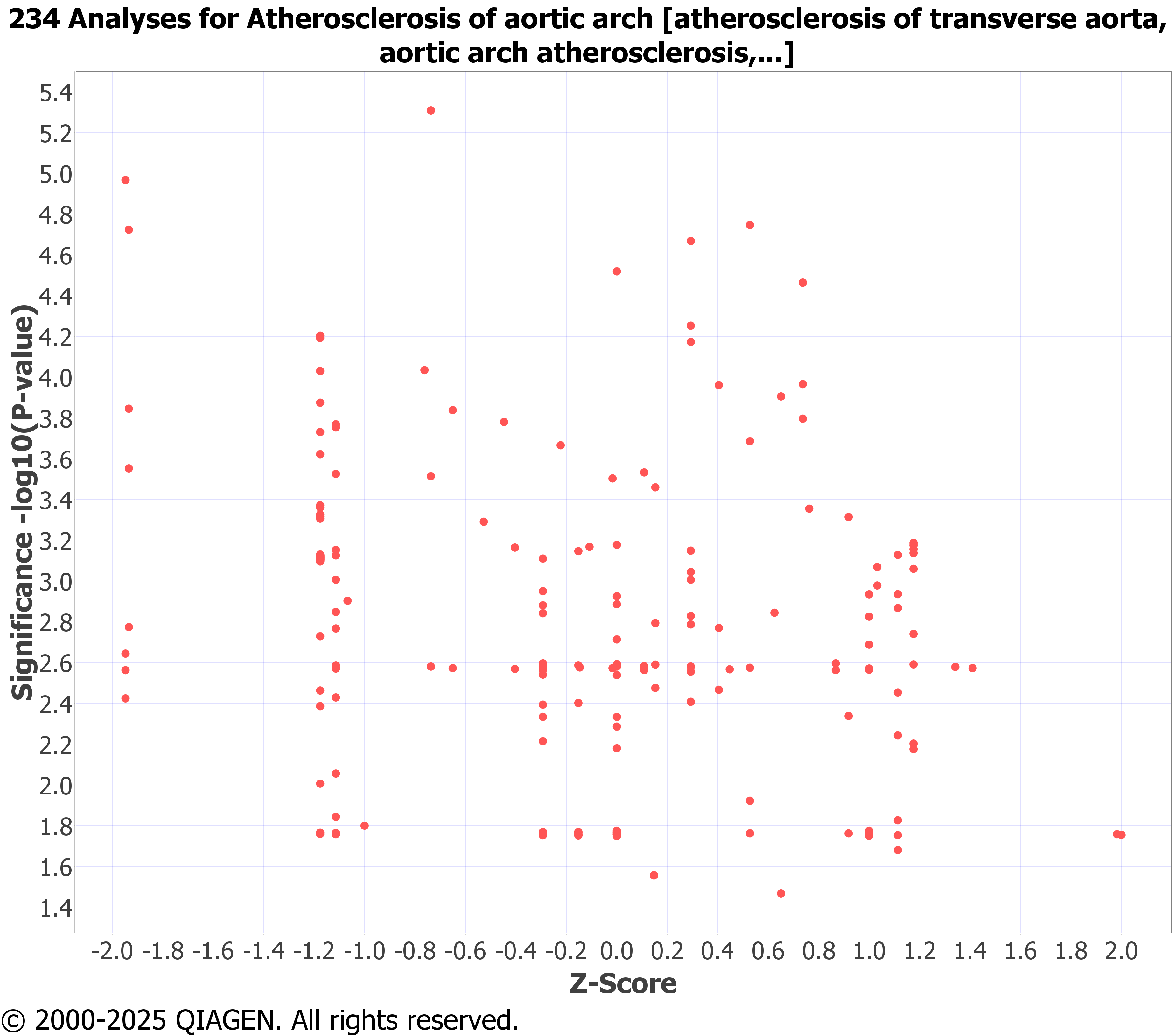

Supplement: Multimedia component 1 [file mmc1.zip › Supplement data/IPA Big data analytics-Atherosclerosis overall signaling and RNA-seq for MerTK expression/234 analyses for AS in aortic arch-1.tif]

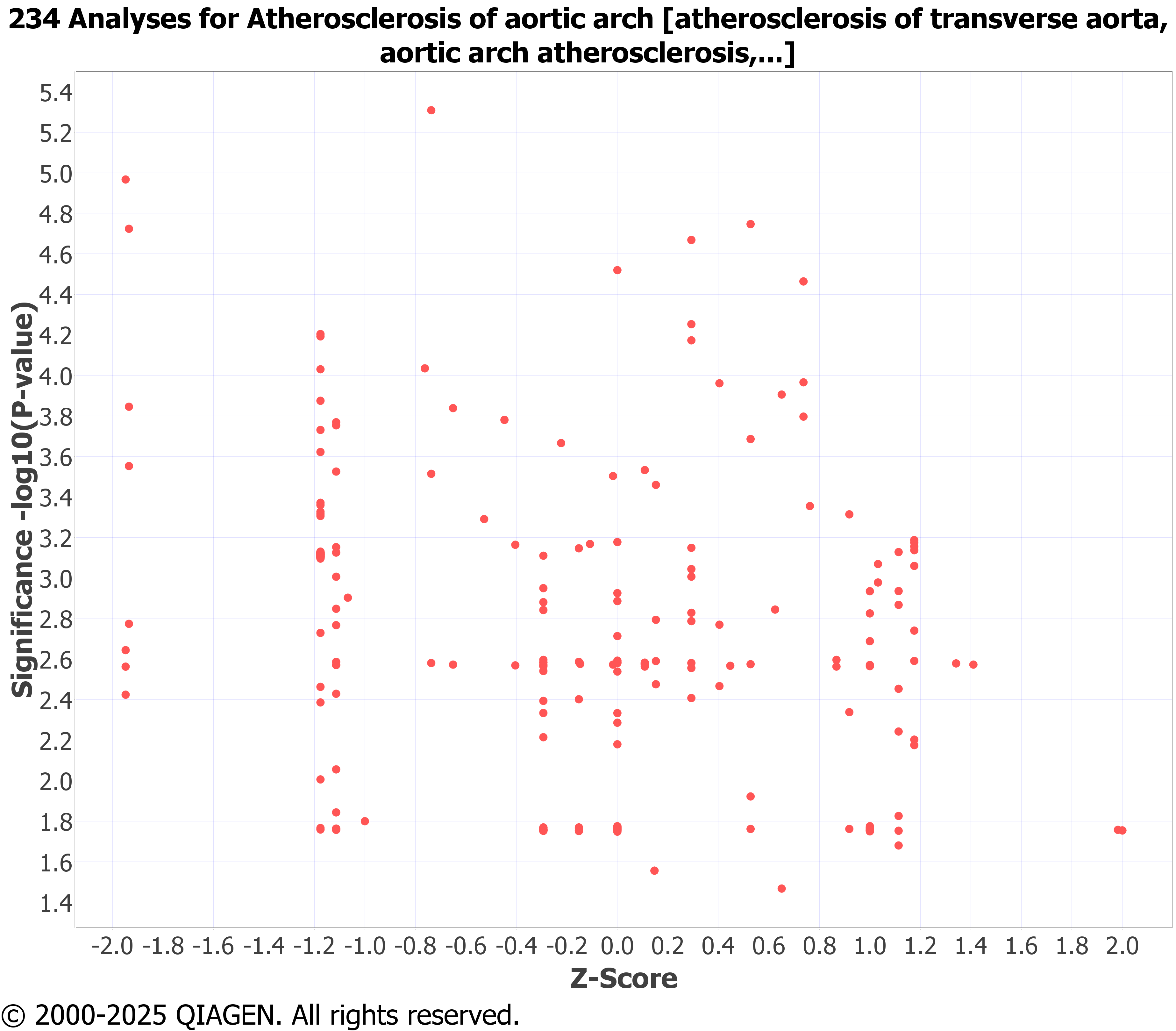

Supplement: Multimedia component 1 [file mmc1.zip › Supplement data/IPA Big data analytics-Atherosclerosis overall signaling and RNA-seq for MerTK expression/234 analyses for AS in aortic arch.tif]

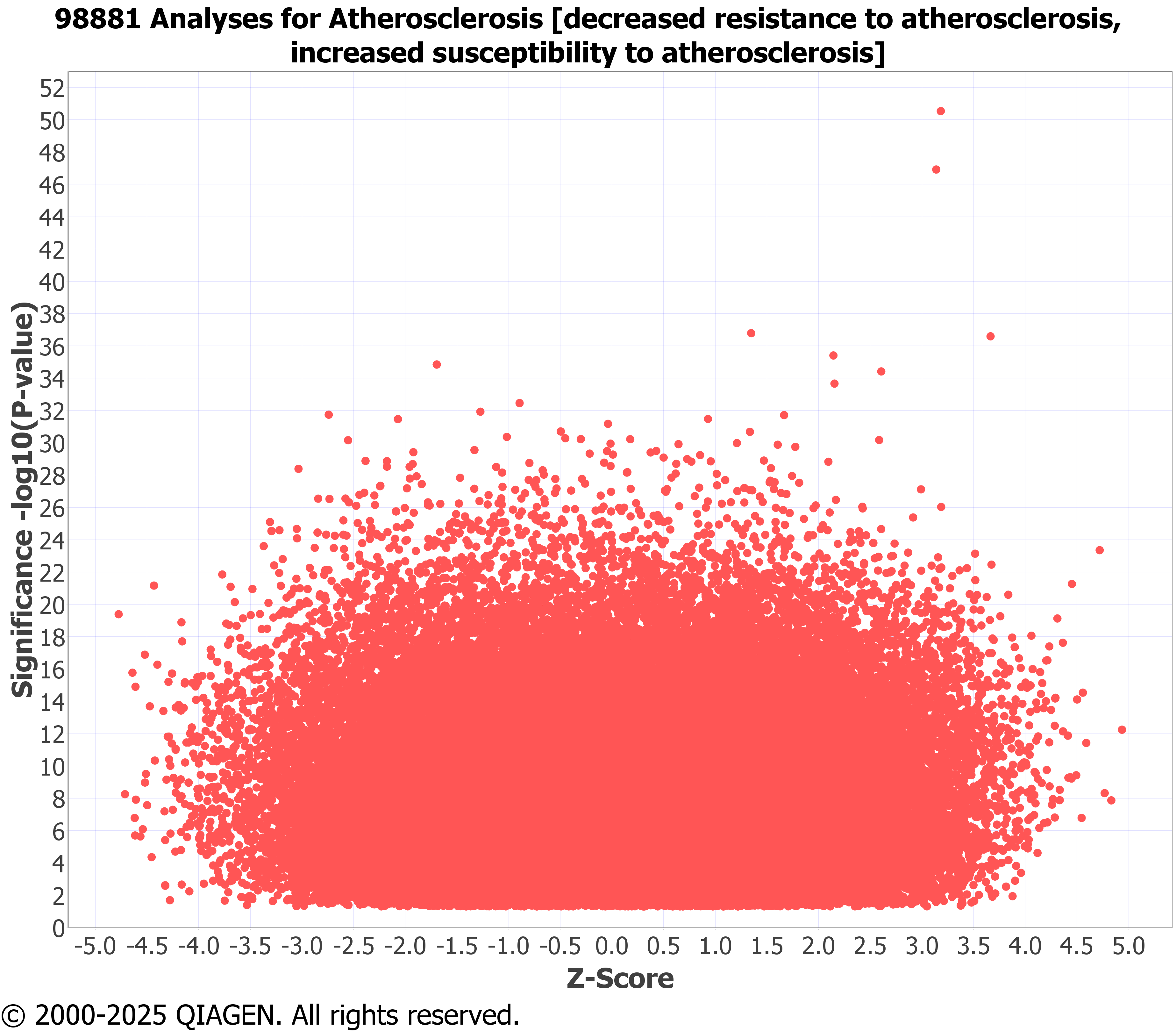

Supplement: Multimedia component 1 [file mmc1.zip › Supplement data/IPA Big data analytics-Atherosclerosis overall signaling and RNA-seq for MerTK expression/98881 analyses for overall atherosclerosis.tif]

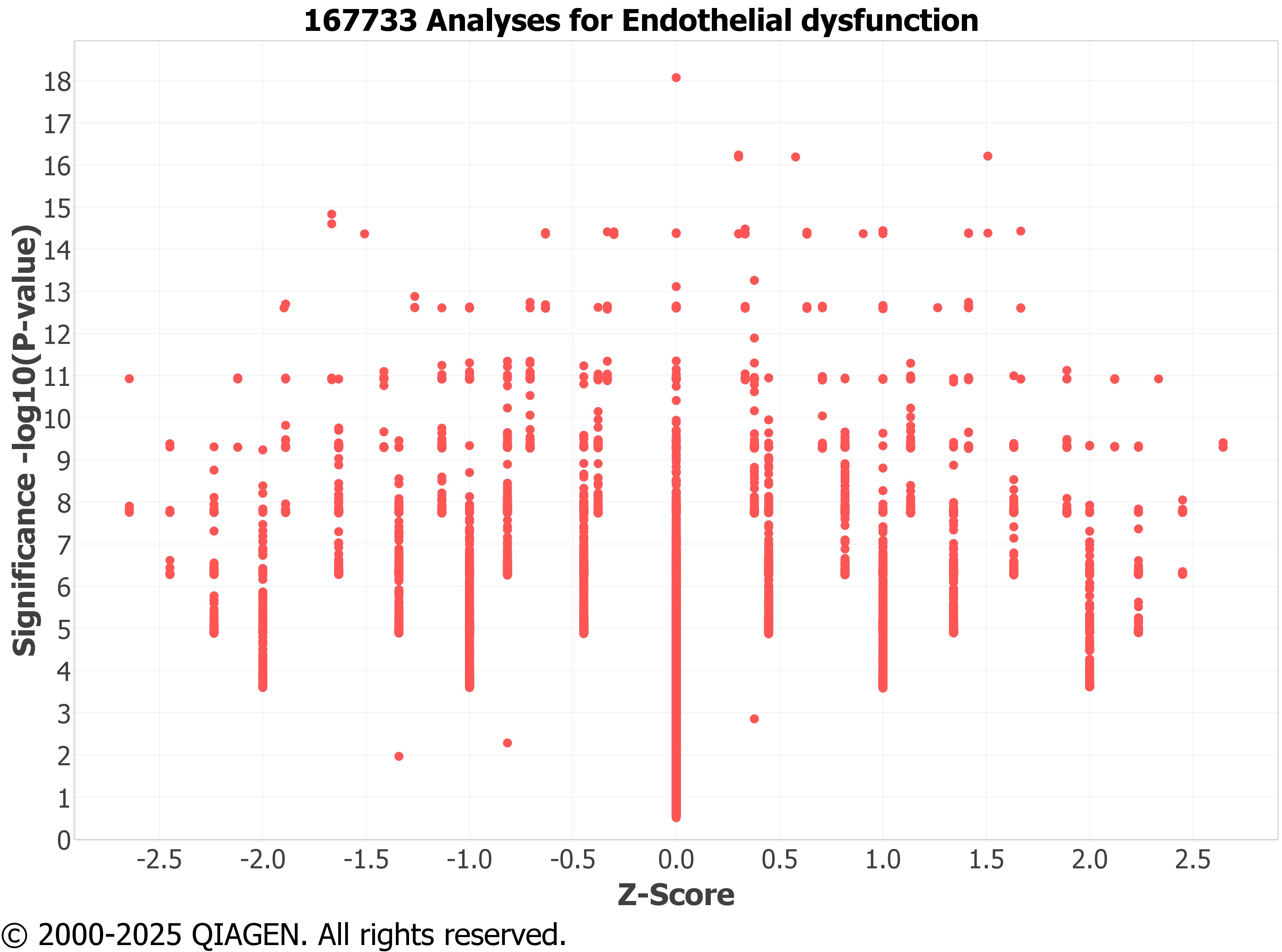

Supplement: Multimedia component 1 [file mmc1.zip › Supplement data/IPA Big data analytics-Endothelial Dysfunction/167733 for endothelial dysfunction.jpg]

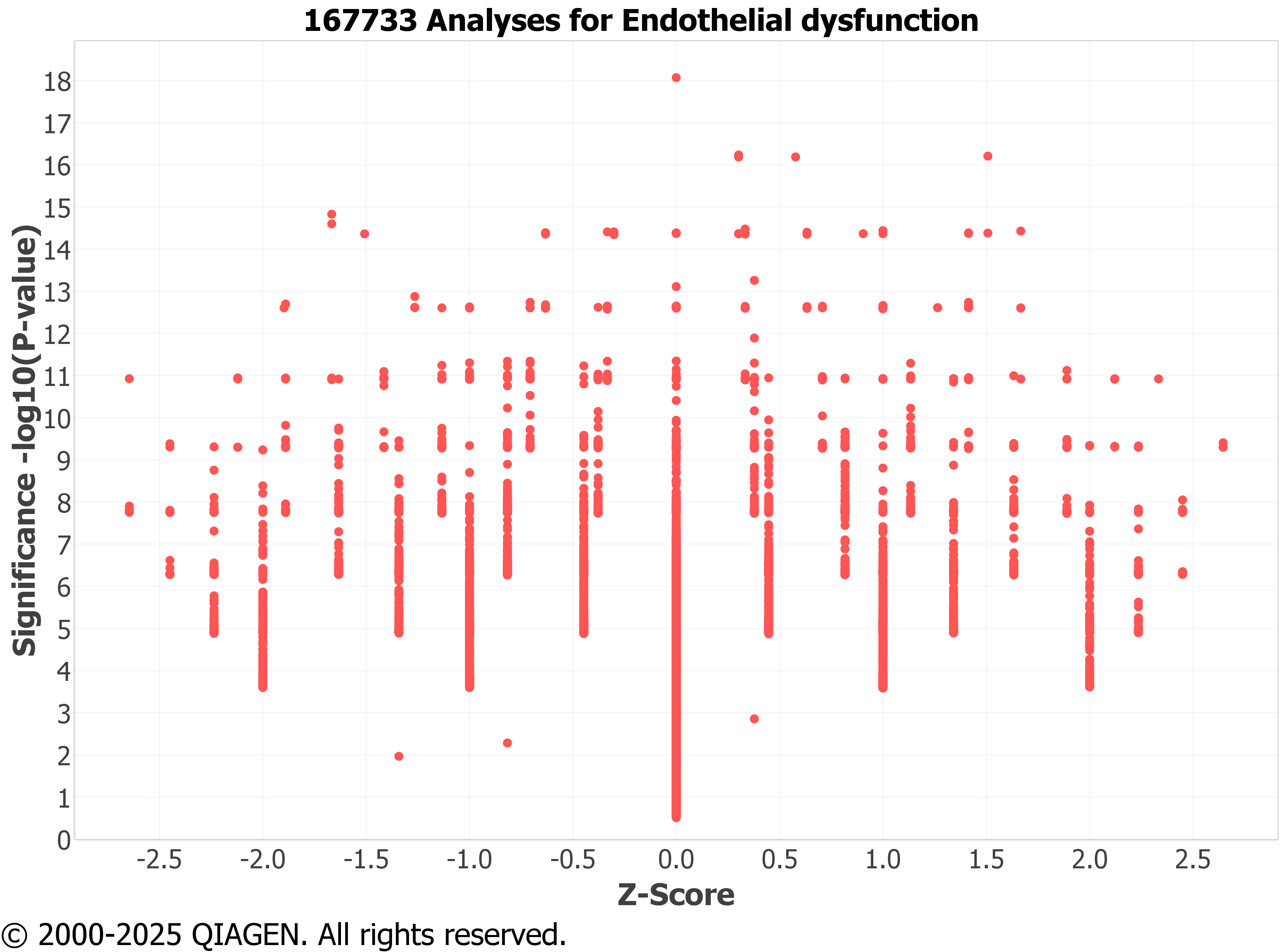

Supplement: Multimedia component 1 [file mmc1.zip › Supplement data/IPA Big data analytics-Endothelial Dysfunction/167733 for endothelial dysfunction.tif]

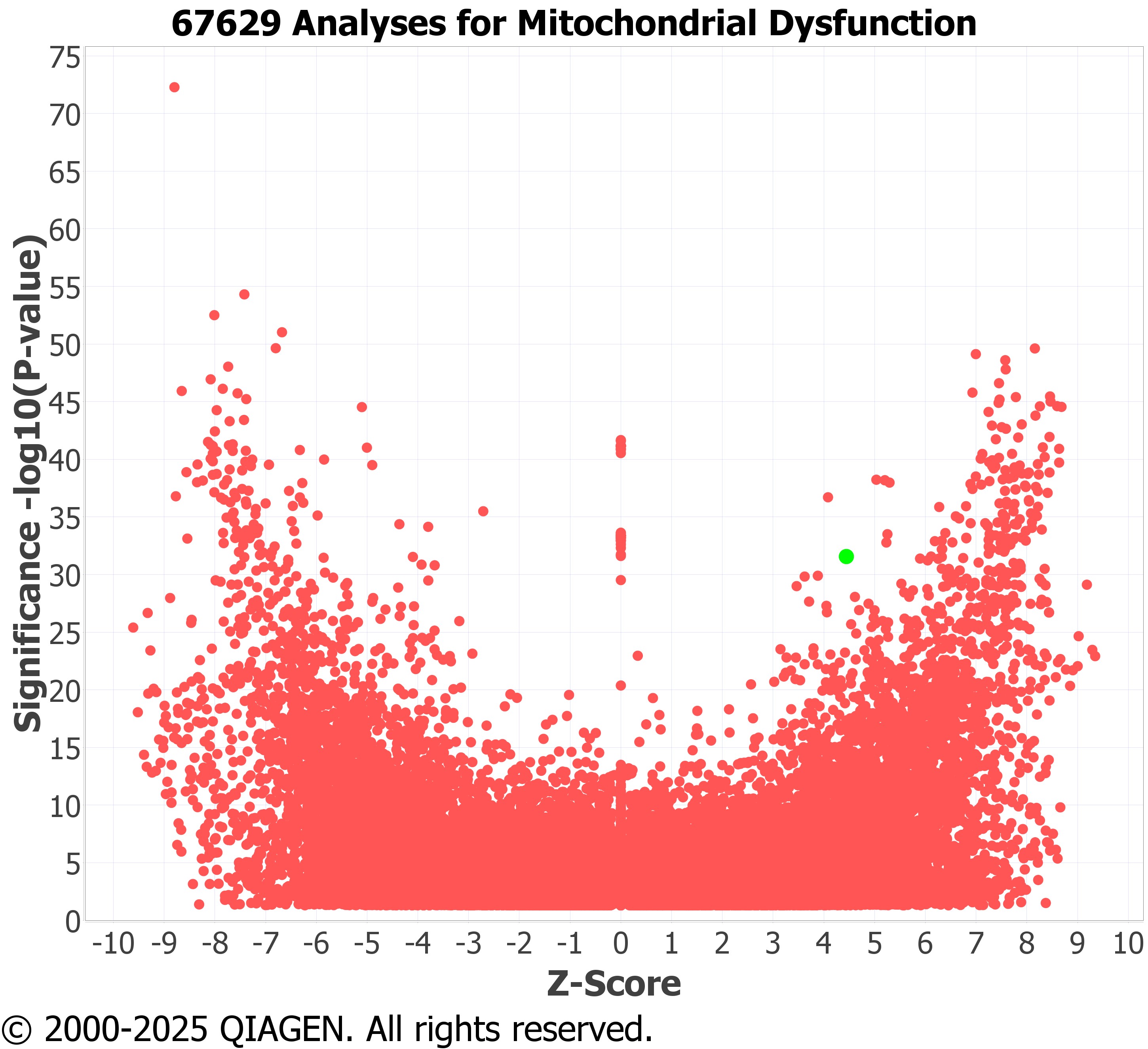

Supplement: Multimedia component 1 [file mmc1.zip › Supplement data/Proteomics in AA/IPA format/IPA/67629 analyses for mitochondrial dysfunction.jpg]

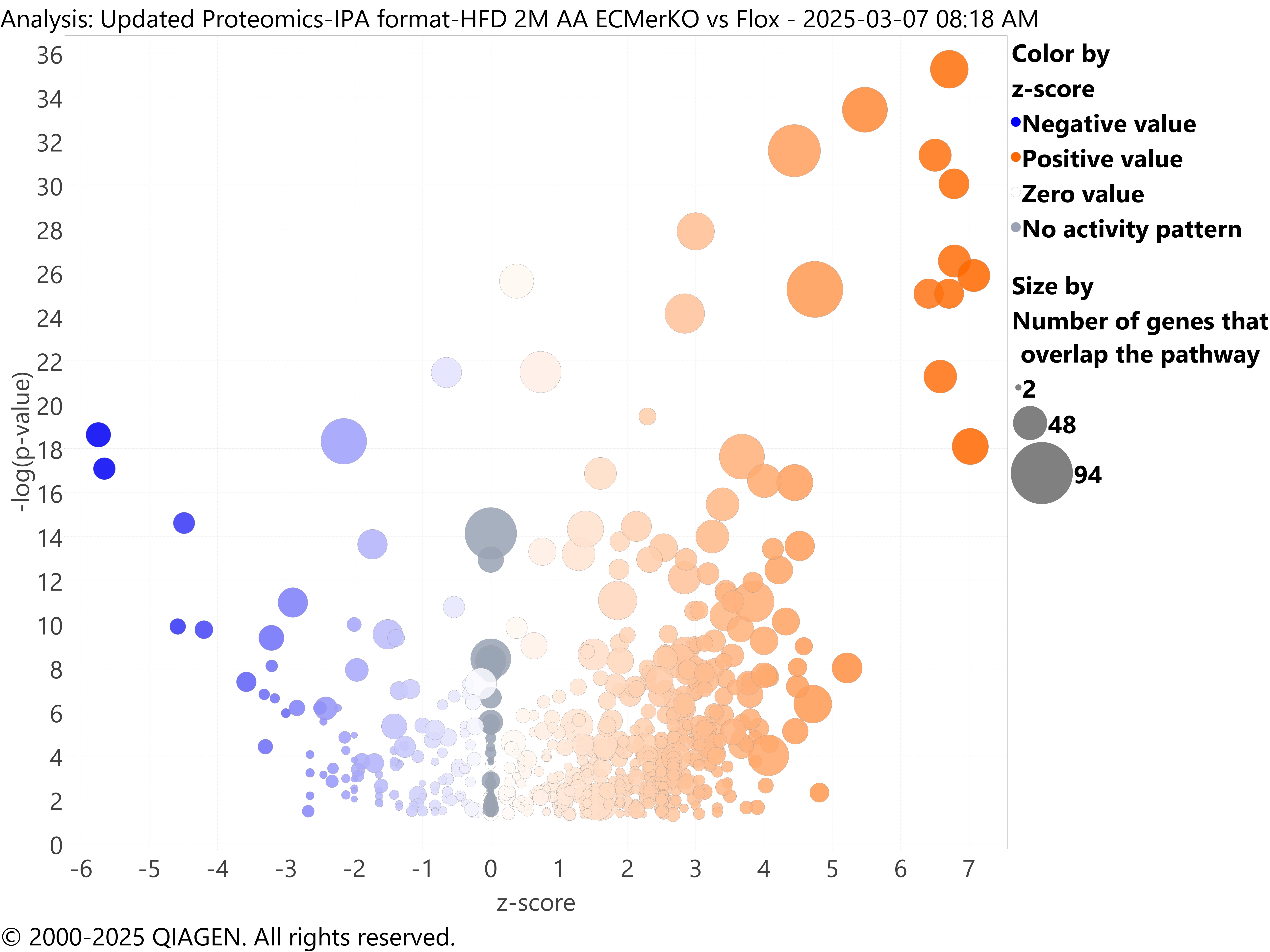

Supplement: Multimedia component 1 [file mmc1.zip › Supplement data/Proteomics in AA/IPA format/IPA/Canoical pathways-Bubble chart (volcano).jpg]

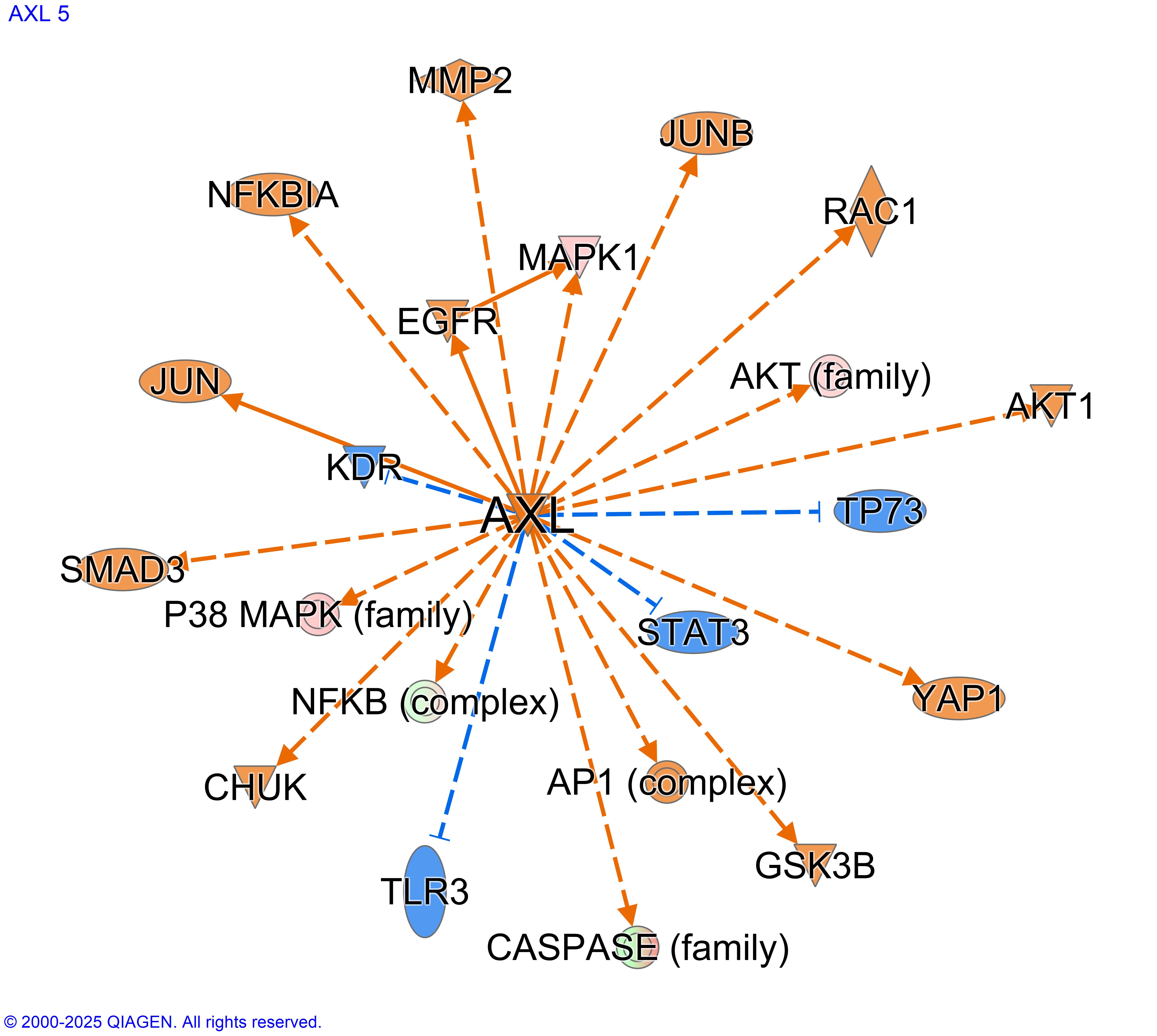

Supplement: Multimedia component 1 [file mmc1.zip › Supplement data/Proteomics in AA/IPA format/IPA/Causal newwork-AXL.jpg]

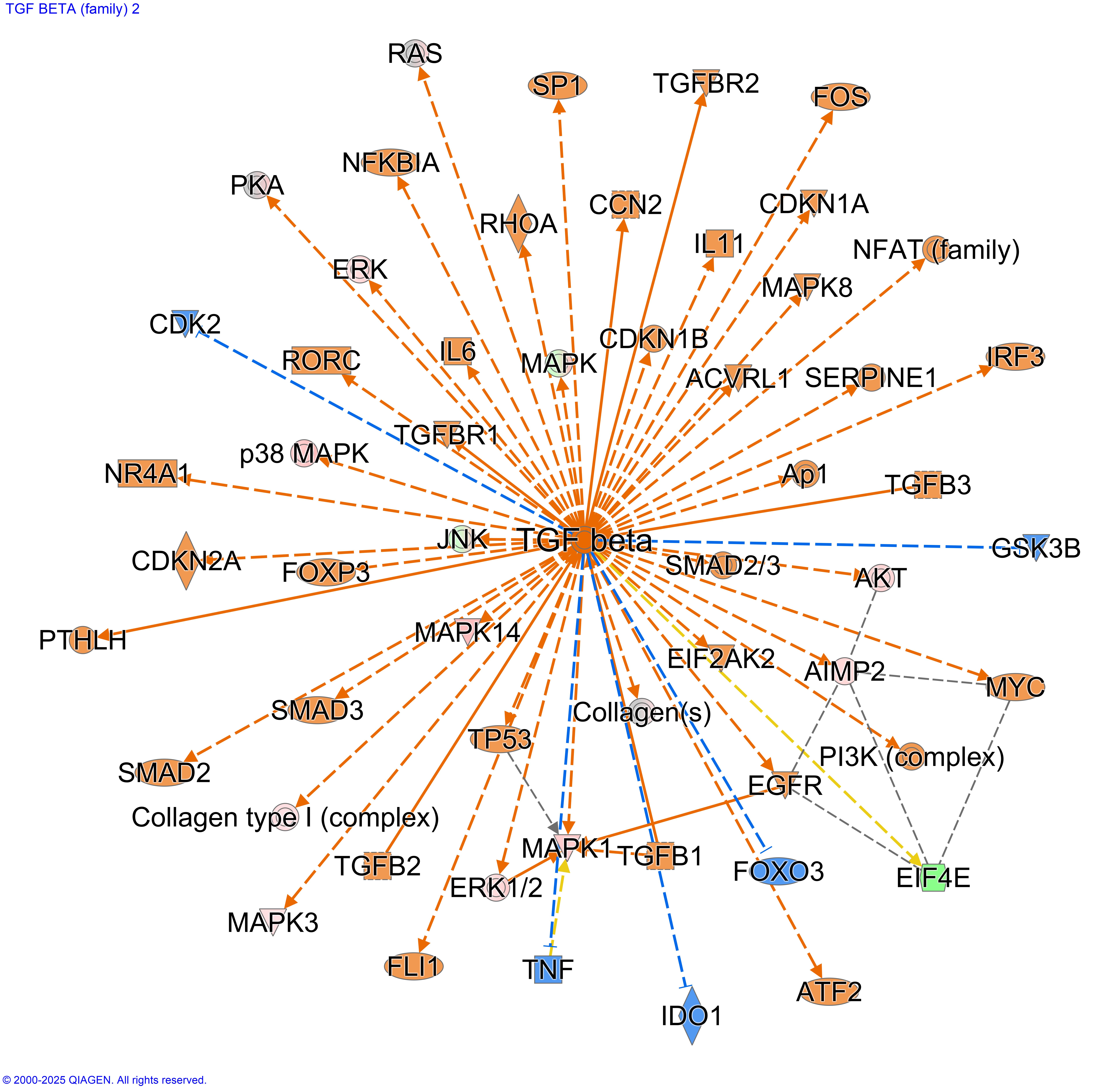

Supplement: Multimedia component 1 [file mmc1.zip › Supplement data/Proteomics in AA/IPA format/IPA/Causal newwork-TGF-beta.jpg]

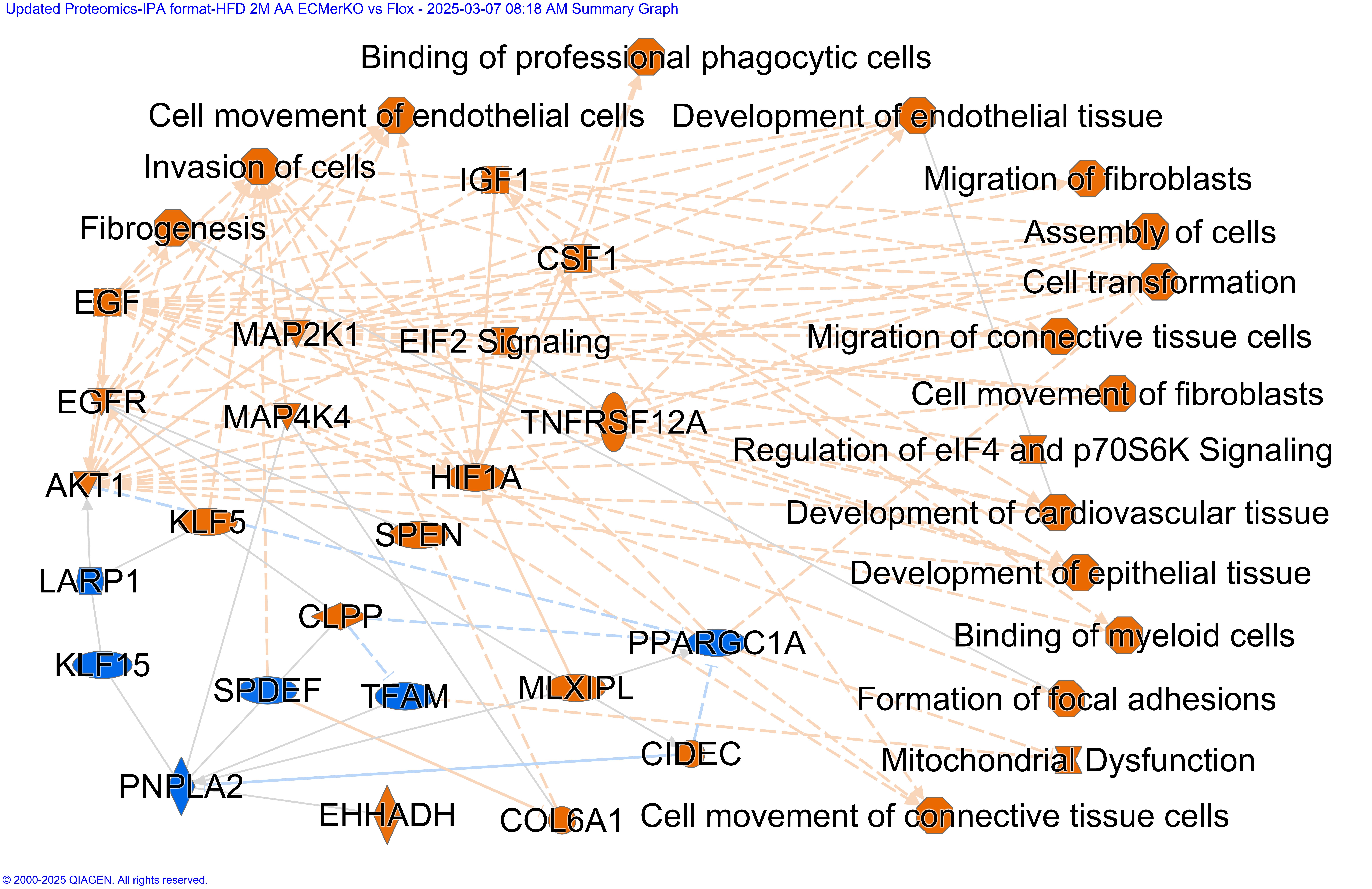

Supplement: Multimedia component 1 [file mmc1.zip › Supplement data/Proteomics in AA/IPA format/IPA/Graphical Summary-1.jpg]

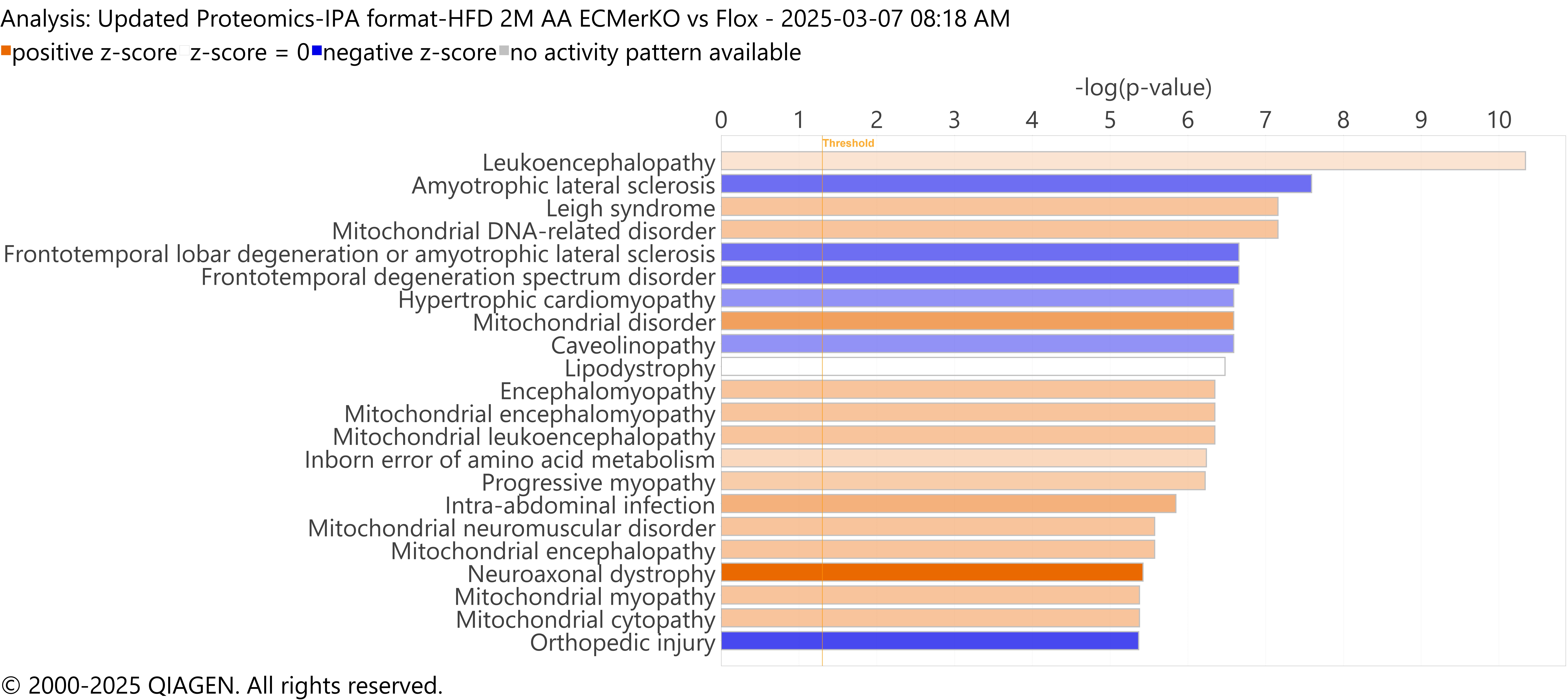

Supplement: Multimedia component 1 [file mmc1.zip › Supplement data/Proteomics in AA/IPA format/IPA/ML Disease Pathways-1.jpg]

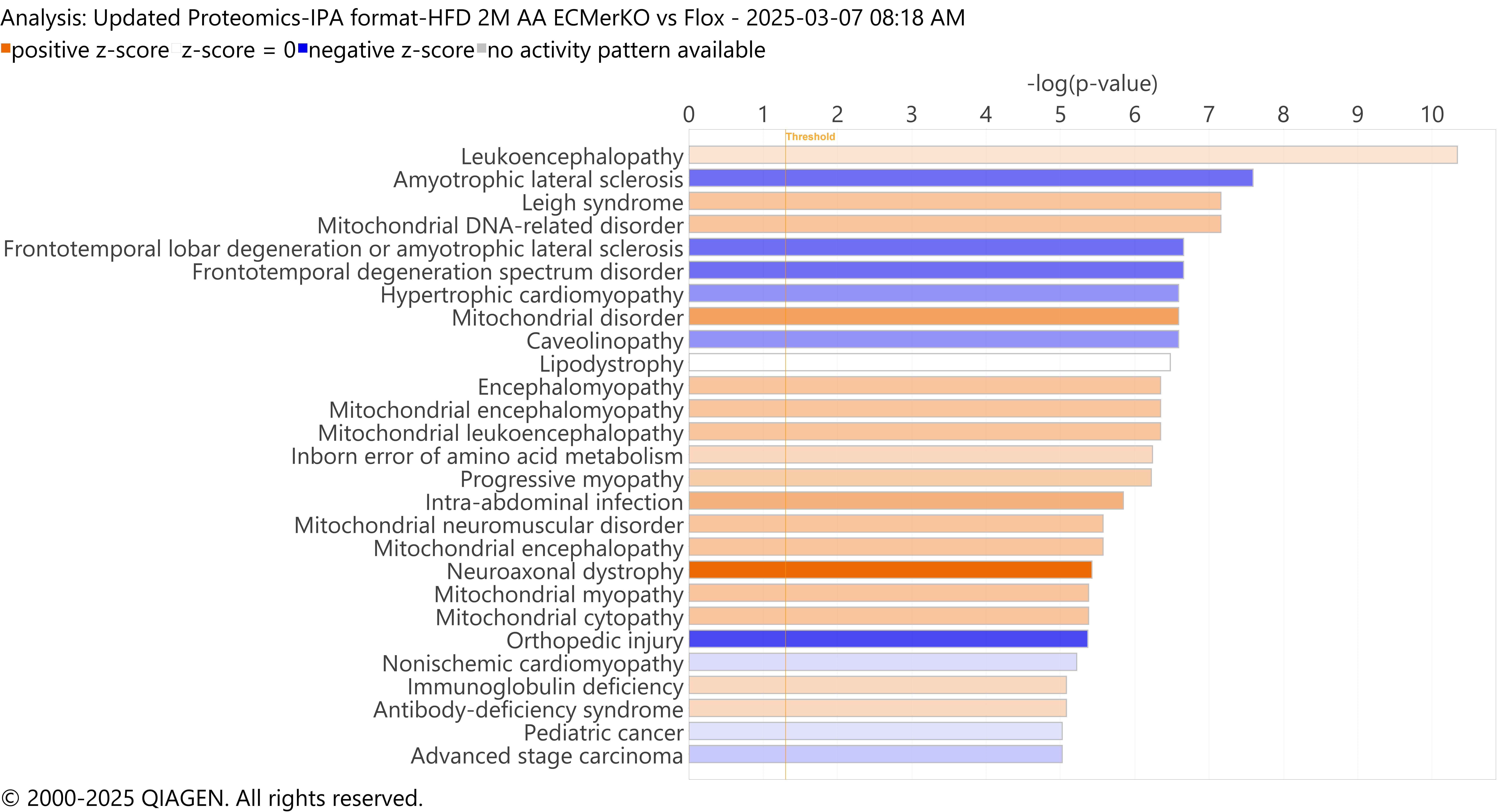

Supplement: Multimedia component 1 [file mmc1.zip › Supplement data/Proteomics in AA/IPA format/IPA/ML Disease Pathways.jpg]
